# Opportunities to improve the quality of inpatient consultation: one hospital’s investigation but an age old struggle

**DOI:** 10.1186/s13584-022-00520-1

**Published:** 2022-01-31

**Authors:** Jennifer P. Stevens, Bruce Landon

**Affiliations:** 1 Center for Healthcare Delivery Science, Beth Israel Deaconess Medical Center, Boston, MA USA; 2 Division for Pulmonary, Critical Care, and Sleep Medicine, Department of Medicine, Beth Israel Deaconess Medical Center, Boston, MA USA; 3 Division for General Medicine, Department of Medicine, Beth Israel Deaconess Medical Center, MA Boston, USA; 4 Department of Healthcare Policy, Harvard Medical School, Boston, MA USA

## Abstract

Inpatient consultation is widely used by hospital physician teams to access specialized expertise and procedures. However, the quality of the resultant consultation varies widely. This commentary describes prior efforts to understand variation in rates of consultation and potential implications across the spectrum of care from underuse to overuse. Improving the quality of consultation requires a full understanding of the aspects of consultation that contribute to quality, including clear requests and communications from the consulting team, but also recognition of organizational and cultural constraints that can impact the availability and quality of consultations provided.

The involvement of specialists in the care of hospitalized patients through the process of consultation is widespread. Within the United States, Medicare beneficiaries hospitalized for medical problems routinely see between one and three specialist consultants when hospitalized and there is substantial variation across regions and even across hospitals within a region [1]. Consultations are used by primary teams to obtain specialized clinical expertise or procedural assistance, though at times they can also be driven by patient or family request. Fundamentally, given the increasing specialization of inpatient care, consultations are essential for patients to effectively access the breadth and depth of medical knowledge. Specialty consultation, however, can also have downsides. Each new physician involved in a case can recommend additional diagnostic and therapeutic interventions that sometimes will not be beneficial to patients and may result in increased length of stay as well as conflicting recommendations. Thus, when considering the quality of consultation, it is important to take a broad view that encompasses all aspects of consultations ranging from the reason for the consultation by the primary team to the timeliness and content of the consult recommendations.

Nearly forty years ago, the “ten commandments” of high quality consults were enumerated by Goldman and colleagues [2]. These commandments included the following: that the consultant should determine the question that is being asked; establish the urgency of the consultation; gather primary data; communicate as briefly as appropriate; make specific recommendations; provide contingency plans; understand her own role in the process; offer educational information; communicate recommendations directly to the requesting physician; and provide appropriate follow-up. While these recommendations were focused on the role of the consultant, Goldman notes, however, that effective consultation is a two-way street requiring effective communication and framing of the consult request from the primary team. Thus, in order understand and improve the quality of consultation, consultants’ thoughts about how they were engaged and involved are also important – was the question asked by the primary team clear and appropriate? Was the clinical situation as urgent as the primary team thought? Finally, beyond the dyad of the primary team/consulting team, an even more holistic view of the quality of consultation would also consider the experience of the patient and family as well as other members of the care team such as nurses, who often must implement the recommendations of consultants [3].

Efforts by our research group and others have drilled down further into exactly what providers mean when they describe a consultation as high quality [4–6]. A primary provider is calling for help from their specialist colleagues when they call for a consultation. To not be heard in one’s cry for help readily feels like a failure to communicate. Thus, critical to a sense of quality is the responsiveness to the primary provider’s sense of urgency. But we also found additional components of high quality of consultation including the consulting team’s (1) decisiveness, (2) thoroughness, (3) level of interest, (4) professionalism, (5) expertise, (6) timeliness, and (7) involvement with the family of the patient. 4.

Our research has found that not every consultation starts solely because of a patient’s clinical need, however, making achieving a high-quality consultation all the more elusive. In the United States, we found that specialist use per admission for elderly patients varies from hospital to hospital and physician to physician [1,7]. For example, a Medicare beneficiary admitted to a hospital in the Northeast of the U.S. is 17% more likely to see a specialist during a hospital stay than in other regions, even after controlling for clinical need. 1 Physicians are known to use resources for a variety of nonclinical reasons, such as local intensity of resource use [8], educational experience or training program patterns of care [9,10], or concerns about malpractice litigation [11]. Such variation in rates of consultations raise concerns about both under- and overuse, which are significant quality of care issues. Suggestive of over-use, we recently found that hospitalists in the US who used specialty consultation more than their colleagues at the same institution used more resources without a difference in patient mortality. Compared with patients treated by other hospitalists, the patients of high-consulting hospitalists also had longer lengths of stay, were less likely to go home, and were more likely to see a specialist within 90 days after discharge, but there was no significant difference in their mortality at 30 days or their likelihood of all-cause readmission. Certainly, underuse is also a concern, particularly in rural settings or small hospitals that might lack readily accessible additional expertise, which may contribute to sizable mortality differences seen between small, rural critical access hospitals in the US as compared with others [12].

Though these tenets were laid out decades ago, formal efforts to measure and improve the quality of inpatient consultation are rare. Importantly, Jarjou’i and colleagues’ recent article in the Israel Journal of Health Policy and Research, “Availability, timeliness, documentation, and quality of consultations among hospital departments: a prospective, comparative study”, describes one such effort at a single large tertiary hospital in Israel [13]. The researchers asked a sample of trainees and attending physicians to evaluate different divisions and departments on their availability, timeliness, and quality of their consultative services. Clear differences stand out between medical and surgical disciplines, with surgeons lagging behind in all three measurable areas of quality. Jarjou’i and colleagues’ evaluation of consultation quality is from the primary perspective of the team caring for the patient and did not ask consultations to rate the quality of the consultation requests they received. They thus supplemented their survey with a small chart review of 300 patient charts to ascertain the quality of the consultation requests.

Jarjou’i et al. elucidate that the use of consultation and the quality of consultation are also dependent on the clinical context, the payment structure, and the hospital and departmental organization. For example, the surgeons in Jarjou’i’s study are largely juggling their operative time against their availability on the clinical floors for patient evaluation. They cannot be in two places at once. In contrast, the more highly rated medical services dedicated a specific individual from each service to handle consultations on a daily basis. Even so, in both cases, respondents describe uneven access to specialty consultation that may arbitrarily give some patients access to specialist care who happen to have proactive providers who call consultations early in the day or who have particularly kind and responsive specialists willing to provide uncompensated use of their time while others do without.

Jarjou’i and colleagues put forward several proposals to improve consultation quality within the context of the Israeli health system, including recommending department-level plans for consult coverage. There also may be other, more generalizable opportunities to improve consultation efficiency and thereby improve access to consultation for patients who truly need a specialist’s evaluation. For example, could e-consults or discussions by phone be sufficient for some clinical circumstances where an exhaustive evaluation by the specialist is not required? [14–16] Are there screening tools that could help primary teams decide if specialty care is necessary or appropriate? [17] Especially in a context where all consultations are not incentivized, there may be opportunities to experiment and test a range of different types of consults, ranging from low-touch telephone reassurance to in-depth specialist evaluation.

What also is striking about Jarjou’i and colleagues’ evaluation of consultation quality is that as with many aspects of health care, it is difficult to improve performance without routine measurement. As Peter Drucker said, “if you can’t measure it, you can’t improve it.” In the United States, virtually all hospitals now have some form of electronic medical record, and this is also the case in Israel and other developed healthcare systems throughout the world. EHRs can be used for electronic ordering of consultations, which can form the backbone of a performance measurement system. At the very least, information on the frequency of consultation from specific teams and the responsiveness and timeliness of recommendations from consulting teams can be collected, and such data might serve as a nidus for more far reaching quality improvement efforts. Routine ascertainment of ratings of aspects of the consultation, both from the perspective of the requesting and consulting teams could be a next step and such data could be form the basis of intuitional efforts to improve the quality of consultations. Such data collection could also be expanded to include the experiences of other members of the care team such as nurses or respiratory therapists who might implement the recommendations of consultants [3].
